# 1604. Predicted Uptake of Novel HIV Treatment Options in the United States

**DOI:** 10.1093/ofid/ofad500.1439

**Published:** 2023-11-27

**Authors:** Elizabeth S Russell, Yan Song, Yipeng Gao, James Signorovitch

**Affiliations:** Merck & Co, Inc, Boston, Massachusetts; Analysis Group, Inc., Boston, Massachusetts, USA, Boston, MA; Analysis Group, Inc., Boston, Massachusetts; Analysis Group, Boston, Massachusetts

## Abstract

**Background:**

People with HIV (PWH) require lifelong treatment to achieve and maintain viral suppression. The modality and frequency of dosing for chronic medications may impact adherence, quality of life, and effectiveness. Preference data reported by potential users can inform which products in development may be preferred among people with various characteristics and social determinants of health, and can inform development for which product may have the largest health impact if it were available on the market. This study used the preference results from a latent class analysis to model uptake by product of a potential future market of HIV treatment options.

**Methods:**

Data from an online discrete choice survey among 829 US adult PWH about treatment modality (daily or weekly oral pills, 3- or 6-monthly injections by health care providers (HCP), or yearly implants) were used in a latent class model to estimate partworth utility of each product in the 4 identified classes of participants. Probabilities of product uptake were estimated based on partworth utility-predicted product use among study participants and weighted for the US population of PWH. Characteristics considered for weighting were those previously reported to be associated with HIV care and treatment outcomes; they were included in weighting if available data from representative national data sources were found through a targeted literature search.

**Results:**

Preference data weighted for the US population of PWH predicts that if all 5 products were available, they would be used in following proportions: 23.8% yearly implants, 23.7% weekly oral pills, 23.6% 6-month HCP-injections, 18.4% daily oral pills, and 10.5% 3-month HCP-injections. Despite oversampling PWH who were < 40 years old, people with a history of food insecurity, and transgender men (Table 1), the weighted model including the characteristics in Table 1 did not alter predicted uptake by more than 2% for any product compared with the unweighted data.

**Figure:**
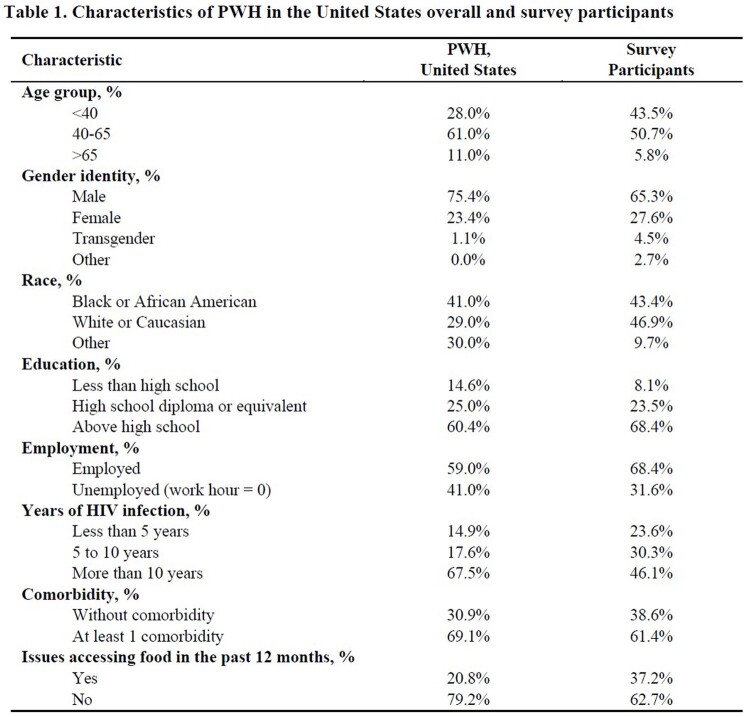

**Conclusion:**

Based on stated preference survey results, if 5 product delivery options for HIV treatment were available, product use would be broadly distributed, with weekly oral pills, yearly implant, and 6-month HCP-injections used in a similar proportion.

**Disclosures:**

**Elizabeth S. Russell, PhD**, Merck & Co. Inc: Employee **Yan Song, PhD**, Merck & Co., Inc.: Grant/Research Support **Yipeng Gao, PhD**, Merck and Co.: Grant/Research Support **James Signorovitch, PhD**, Merck & Co., Inc.: Grant/Research Support

